# The impact of sleep–wake problems on health-related quality of life among Japanese nursing college students: a cross sectional survey

**DOI:** 10.1186/s12955-022-02063-0

**Published:** 2022-11-10

**Authors:** Mai Adachi, Yuki Nagaura, Hiromi Eto, Hideaki Kondo, Chiho Kato

**Affiliations:** 1 Graduate School of Biomedical Sciences, Nagasaki University, Jikei Hospital, NagasakiKumamoto, Japan; 2 Department of General Medicine, Institute of Biomedical Sciences, Nagasaki University, Nagasaki, Japan; 3 Department of Reproductive Health, Institute of Biomedical Sciences, Nagasaki University, Nagasaki, Japan; 4 Sleep Center, Social Medical Corporation Shunkaikai, Inoue Hospital, Nagasaki, Japan; 5 International Institute for Integrative Sleep Medicine, University of Tsukuba, Ibaraki, Japan; 6 Japanese Red Cross College of Nursing, Tokyo, Japan

**Keywords:** Insomnia, Mental health, Quality of life, Sleep duration, Total sleep time

## Abstract

**Aim:**

This study was conducted to examine the impact of sleep–wake problems on health-related quality of life of Japanese nursing college students.

**Methods:**

This cross-sectional study was conducted in 2019 on 150 third and fourth-year nursing college students from two locations in Japan. Insomnia severity was assessed using the Insomnia Severity Index (ISI) and health-related quality of life using the SF-8 questionnaire. The total sleep time (TST) was divided into 3 groups: < 6 h, 6–7 h (reference), and ≥ 7 h. The total ISI score was divided into 2 groups: ≥ 8 points and < 8 points (reference). Logistic regression analysis was performed to evaluate sleep–wake problems related to decline in mental health.

**Results:**

The median mental health indicated in the SF-8 questionnaire was divided into two groups, and the factors causing decline in mental health were investigated. The odds ratios (95% confidence interval) for adjusted ISI ≥ 8 and TST on weekdays < 6 h was 6.51 (2.96–14.30) and 3.38 (1.40–8.17), respectively. Mental health status was significantly lower when ISI ≥ 8 and even lower when TST < 6 h.

**Conclusion:**

Insomnia and short sleep duration are associated with decreased mental health status in nursing college students. Many tended to lack sleep on weekdays. Sleep–wake problems identified while in university should be comprehensively dealt with.

## Introduction

Sleep pattern among college students undergoes changes as part of their physiological development leading to adulthood. The power of slow-wave sleep decreases after puberty, around the age of 12 years [1], and a phase delay of the circadian rhythm of 1 to 3 h occurs [2]. This delay peaks around the age of 15 to 20 years and moves forward thereafter [3]. For a while after becoming university students, it corresponds with the time when the phase is the most backward, and before graduation, it is the time when the phase is about to move forward. As for the morning or evening chronotype, the ratio of evening chronotype was 6.9% in first-year university students in China (*N* = 4,531, average age 19.2 years) [4] and 21.9% in another report involving Italian and Spanish students (*N* = 2,135, average age 22.2 years) [5].

In addition to changes in physiological sleep–wake cycle, college students are exposed to changes in their living environment and habits associated with becoming a university student. As such, subjective deterioration in sleep quality is as high as 50–60% [6–8]. This causes sleep–wake problems. In modern society, light exposure associated with the use of smartphones and internet browsing until late at night also intensifies this tendency [9, 10]. Despite regression of the sleep phase, students are required to wake up early in the morning to go to school and also participate in other social activities. There are concerns about sleep deprivation and problems associated with social jet lag due to sleep deprivation and catch-up sleep during holidays [11–13]. It has been reported that absenteeism increases and academic achievement declines owing to delayed sleep–wake phase disorder that has reached a more pathological situation or when insufficient sleep syndrome coexists with delayed sleep–wake phase disorder [14–16].

The prevalence of insomnia in college students has been reported to be 22.1–38.6% [14, 17, 18]. However, these results were obtained from a questionnaire and may vary according to the cutoff value used and the diagnostic algorithm, but the prevalence is expected to decrease with a more detailed questionnaire [19]. However, the presence of sleep deprivation may mask the symptoms of insomnia due to the accumulation of sleep debt. On the contrary, when delayed sleep–wake phase disorder manifests, difficulty initiating and maintaining sleep become evident, which necessitates differentiation from insomnia. Due circumspection should be taken in diagnosing insomnia, as sleep insufficiencies and delayed sleep phase tendencies may coexist [11, 14].

Many reports have shown that sleep–wake problems in college students are associated with depressive symptoms. Poor sleep quality [8, 20], evening chronotype [4], insomnia [17, 18], insufficient sleep syndrome [17], and sleep debt [21] are all associated with depressive symptoms. Insomnia during college also increases the risk of subsequent depression [22]. Additionally, a survey of newly graduated nurses reported that pre-employment sleep problems at the time of employment were associated with subsequent turnover [23, 24]. Intervening in sleep–wake problems identified before graduating from university is important to ensure that individuals continue working healthily after graduation.

In order to examine the impact of sleep–wake problems on health-related quality of life (HRQOL) of Japanese nursing college students, we evaluated the relationship between subjective sleep quality, severity of insomnia, chronotype, and HRQOL among third- and fourth-year students enrolled in nursing colleges.

## Methods

### Participants, study design, and ethical consideration

A total of 212 third-year students and 216 fourth-year students from 2 nursing colleges in Japan were invited to participate in the study. This survey was conducted from November to December 2019. The participants accessed the Google Form from the QR code in the manual using their smartphone and answered the questionnaire online. This study was reviewed by the author's belonging university and was carried out after obtaining permission (permission number: 19091203–2). The research plan was explained verbally using the instruction manual, and those from whom consent could be obtained were selected as the study participants.

### Measures

Regarding participants’ background, we surveyed their age, sex, living arrangement (living alone/living with other people), exercise habits, drinking habits, smoking status (smokers, ex-smokers, and non-smokers), breakfast, night time meals after dinner until bedtime as well as use of mobile devices during bedtime. Habitual drinkers were defined as those who drink at least thrice a week. Those with exercise habits were defined as people who exercise at least twice a week. Based on the respondent’s answer to the question “Are you a morning or an evening person?” the participants were divided into four groups: 1. definite morning type, 2. moderate morning type, 3. moderate evening type, and 4. definite evening type. Subjective sleep quality, insomnia severity, and HRQOL was assessed using the questionnaire.

### Pittsburgh Sleep Quality Index (PSQI)

Subjective sleep quality was assessed using the PSQI, which has 9 items. This questionnaire consists of seven subscales (sleep quality, sleep latency, sleep duration, sleep efficiency, sleep disturbances, hypnotic use, daytime dysfunction). Each subscale is scored based on a 4-point scale (0, 1, 2, and 3 points). The total score ranges from 0 to 21 points, with higher scores indicating more impaired sleep. The cutoff score, which indicates the pathological level, is 6 points (Buysse et al., 1989; Doi et al., 2000). The percentage of those with ≥ 2 points on each subscale and the percentage of those with total scores of ≥ 6 points were calculated. In this study, we asked questions about bedtime, wake time, and sleep onset latency by dividing them into weekdays and weekends. The time in bed (TIB), total sleep time (TST), and sleep efficiency were calculated from the time and time information. The TST was divided into 3 groups of < 6 h, 6–7 h, and ≥ 7 h. Catch-up sleep was calculated from TST on weekends and weekdays: Catch-up sleep = Holiday TST – Weekday TST. The percentage of those who had catch-up sleep of ≥ 2 h was also calculated.

### Insomnia Severity Index (ISI)

The severity of insomnia was assessed using ISI, which has 7 items. The global score of this index ranges from 0 to 28 points, with higher scores indicating greater insomnia severity [25]. The total score is interpreted defined as follows: 0–7 = no clinically significant insomnia, 8–14 = subthreshold insomnia, 15–21 = moderate clinical insomnia, and 22–28 = severe clinical insomnia. The cutoff score, which indicates the pathological level, was 10 points. The percentage of those with ISI total scores of ≥ 8 and ≥ 10 was calculated. We also calculated the percentage of insomnia symptoms. The severity of difficulty initiating sleep, difficulty maintaining sleep, and early waking are answered as “none”, “mild”, “moderate”, “severe”, or “very severe”. Responders who had “mild” to “very severe” symptoms were defined as having difficulty initiating sleep, difficulty maintaining sleep, and early waking.

### SF-8

HRQOL was evaluated using the SF-8 questionnaire, which has 11 items. Eight subscales (physical functioning, role limitations because of poor physical health (role physical), bodily pain, general health perception, vitality, social functioning, role limitations due to poor emotional health (role emotional), and mental health) were scored based on the 2007 national standard values. The national standard value was set to 50 points, and the standard deviation was set to 10 points [26]. Two summary scores, physical component summary (PCS) and mental component summary (MCS), were calculated based on the subscales.

### Data analysis

Statistical processing was performed using R version 4.1.2 and EZR version 1.55 [27]. The continuous variables were shown in median (IQR) because normality was not observed. The Mann–Whitney U test was used for comparison between two groups of continuous variables. The Kruskal–Wallis test was used for comparison between three or more groups, and the Bonferroni method was used for the post-hoc test. The correlation of continuous variables was evaluated by Spearman's *ρ*. Fisher's exact test was used to test the independence of nominal variables. The significance probability was 0.05.

To clarify the factors related to sleep and wakefulness that affect the decrease in MCS of SF-8, logistic regression analysis was performed by dividing the subjects into two groups by the median MCS. A univariate analysis was performed to determine the odds ratio (95% confidence interval) adjusted for sex, year level, smoking habits, and habitual drinking from the following items that were significant in the univariate analysis: weekday sleep time (< 6 h, 6–7 h (reference: ref), ≥ 7 h), and insomnia severity (ISI ≥ 8 vs. ISI < 8 (ref)).

## Results

Consent was obtained from 150 out of 428 students (35.0%). There were 142 women (94.7%) with a median age (IQR) of 22 (21–22) years. There were 72 (48%) participants with PSQI ≥ 6, all of whom were female. The definite evening type was 43 (28.7%) and the moderate evening type was 57 (38.0%). The number of people with ISI ≥ 8 or ISI ≥ 10 was 43 (28.7%) and 23 (15.3%), respectively. Twenty-seven (18.0%) had difficulty initiating sleep, difficulty maintaining sleep, or early-morning awakening. Late sleep and late waking-up tendencies were evident on weekends, and 60 (40%) of them slept for more than 2 h longer than weekdays on weekends (Table 1). The shorter the total sleep time on weekdays, the longer the catch-up sleep on weekends (Spearman’s ρ = -0.57, *P* < 0.001).

**Table 1:** Demographic and sleep characteristics

N	150
Age years, median (IQR)	22 (21–22)
Third grade/Fourth grade	62/88
Women, N (%)	142 (94.7)
Living alone, N (%)	100 (66.7)
Non-breakfast eater, N (%)	37 (24.7)
Night snacker, N (%)	65 (43.3)
Smoking	
Never, N (%)	143 (95.3)
Present, N (%)	3 (2.0)
Ex-smoker, N (%)	4 (2.7)
Habitual drinking, N (%)	22 (14.7)
Regular physical exercise, N (%)	28 (18.7)
Cell-phone use in bed, N (%)	136 (90.7)
PSQI, median (IQR)	5.0 (4.0–7.0)
PSQI ≥ 6, N (%)	72 (48.0)
Evening Chronotype, N (%)	100 (66.7)
ISI, median (IQR)	5.0 (2.0–8.0)
ISI ≥ 8, N (%)	43 (28.7)
ISI ≥ 10, N (%)	23 (15.3)
Weekday TST hr, median (IQR)	6.33 (5.50–7.31)
< 6 h, N (%)	58 (38.7)
6–7 h, N (%)	39 (26.0)
≥ 7 h, N (%)	53 (35.3)
Weekend TST hr, median (IQR)	8.00 (7.00–9.00)
< 6 h, N (%)	12 (8.0)
6–7 h, N (%)	21 (14.0)
≥ 7 h, N (%)	117 (78.0)
Catch-up sleep, median (IQR)	1.50 (0.50–2.75)
≥ 2 h, N (%)	60 (40.0)

*IQR* interquartile range, *ISI* Insomnia severity index, *PSQI* Pittsburgh sleep quality index, *TST* Total sleep time

When the sleep time on weekdays was divided into 3 groups, the sleep time was short among the third-year students, the PSQI total score was high in the group with < 6 h sleep due to the influence of short sleep, and the chronotype was evening type. In the group with short sleep time on weekdays, they tended to sleep late and get up early, and most of them slept for ≥ 2 h longer than weekdays on holidays. The MCS of the SF-8 tended to decrease as the sleep time on weekdays became shorter, but no significant difference was observed in the post-hoc test (Table 2). Comparing the 2 groups, ISI ≥ 8 and ISI < 8, the ISI ≥ 8 group had more difficulty initiating and maintaining sleep, but no significant difference was observed in TST. Regarding HRQOL, the decrease in MCS was remarkable in the ISI ≥ 8 group (Table 3).

**Table 2:** Comparisons of sleep characteristics and health-related quality of life between weekday total sleep time

	< 6 h	6–7 h	≥ 7 h	*P* value
N	58	39	53	
Third grade/Fourth grade	35/23	14/25	13/40	< 0.001
PSQI, median (IQR)	6.0 (5.0–8.0)	5.0 (3.5–6.5)	4.0 (2.0–6.0)	< 0.001
PSQI ≥ 6, N (%)	38 (65.5)	16 (41.0)	18 (34.0)	0.002
Evening Chronotype, N (%)	47 (81.0)	21 (53.8)	32 (60.4)	0.009
ISI, median (IQR)	5.0 (3.0–8.0)	6.0 (2.5–9.0)	4.0 (1.0–8.0)	0.35
ISI ≥ 8, N (%)	16 (27.6)	12 (30.8)	15 (28.3)	0.95
SI ≥ 10, N (%)	9 (15.5)	7 (17.9)	7 (13.2)	0.78
Difficulty initiating sleep, N (%)	8 (13.8)	6 (15.4)	10 (18.9)	0.78
Difficulty maintaining sleep, N (%)	2 (3.4)	2 (5.1)	2 (3.8)	1
Early waking, N (%)	3 (5.2)	1 (2.6)	2 (3.8)	0.88
Weekday TST hr, median (IQR)	5.39 (4.83–5.67)	6.42 (6.00–6.50)	7.75 (7.17–8.33)	< 0.001
Weekend TST hr, median (IQR)	7.83 (6.50–8.96)	7.83 (7.00–8.83)	8.50 (7.75–9.42)	0.03
< 6 h, N (%)	7 (12.1)	1 (2.6)	4 (7.5)	0.003
6–7 h, N (%)	13 (22.4)	7 (17.9)	1 (1.9)	
≥ 7 h, N (%)	38 (65.5)	31 (79.5)	48 (90.6)	
Catch-up sleep, median (IQR)	3.00 (1.00–4.13)	1.50 (0.50–2.17)	0.50 (0.00–1.50)	< 0.001
≥ 2 h, N (%)	39 (67.2)	15 (38.5)	6 (11.3)	< 0.001
SF8				
Physical functioning, median (IQR)	54.3 (54.3–54.3)	54.3 (54.3–54.3)	54.3 (54.3–54.3)	0.05
Role physical, median (IQR)	54.9 (47.8–54.9)	54.9 (45.7–54.9)	54.9 (54.9–54.9)	0.03
Bodily pain, median (IQR)	59.1 (51.3–59.1)	59.1 (51.3–59.1)	59.1 (51.3–59.1)	0.87
General health, median (IQR)	52.7 (43.0–52.7)	52.7 (52.7–60.0)	52.7 (52.7–60.0)	0.06
Vitality, median (IQR)	55.5 (47.4–55.5)	55.5 (47.4–55.5)	55.5 (47.4–55.5)	0.40
Social functioning, median (IQR)	55.2 (47.3–55.2)	55.2 (47.3–55.2)	55.2 (47.3–55.2)	0.59
Role emotional, median (IQR)	49.0 (43.7–55.2)	49.0 (49.0–55.2)	49.0 (49.0–55.2)	0.13
Mental health, median (IQR)	45.1 (38.3–51.2)	51.2 (45.1–51.2)	51.2 (45.1–56.7)	0.02
PCS, median (IQR)	53.8 (51.7–55.5)	52.1 (48.6–55.5)	54.6 (51.5–56.2)	0.38
MCS, median (IQR)	46.3 (40.2–51.7)	48.7 (45.9–54.2)	50.0 (44.7–54.5)	0.04

*IQR* interquartile range, *ISI* Insomnia severity index, *MCS* Mental component summary, *PCS* Physical component summary, *PSQI* Pittsburgh sleep quality index, *TST* total sleep time

**Table 3:** Comparisons of sleep characteristics and health-related quality of life between ISI < 8 group and ISI ≥ 8 group

	ISI < 8	ISI ≥ 8	*P* value
N	107	43	
Third grade/Fourth grade	45/62	17/26	0.86
PSQI, median (IQR)	5.0 (3.0–6.0)	8.0 (6.0–9.0)	< 0.001
PSQI ≥ 6, N (%)	39 (36.4)	33 (76.7)	< 0.001
Evening Chronotype, N (%)	68 (63.6)	32 (74.4)	0.25
ISI, median (IQR)	3.0 (2.0–5.0)	10.0 (9.0–12.0)	< 0.001
Difficulty initiating sleep, N (%)	5 (4.7)	19 (44.2)	< 0.001
Difficulty maintaining sleep, N (%)	0 (0.0)	6 (14.0)	< 0.001
Early waking, N (%)	2 (1.9)	4 (9.3)	0.06
Weekday TST hr, median (IQR)	6.33 (5.50–7.25)	6.33 (5.50–7.12)	0.66
< 6 h, N (%)	42 (39.3)	16 (37.2)	0.95
6–7 h, N (%)	27 (25.2)	12 (27.9)	
≥ 7 h, N (%)	38 (35.5)	15 (34.9)	
Weekend TST hr, median (IQR)	8.00 (7.00–9.00)	8.00 (7.00–8.67)	0.38
< 6 h, N (%)	9 (8.4)	3 (7.0)	0.85
6–7 h, N (%)	14 (13.1)	7 (16.3)	
≥ 7 h, N (%)	84 (78.5)	33 (76.7)	
Catch-up on sleep, median (IQR)	1.50 (0.50–2.88)	1.00 (0.42–2.54)	0.56
≥ 2 h, N (%)	46 (43.0)	14 (32.6)	0.27
SF8			
Physical functioning, median (IQR)	54.3 (54.3–54.3)	54.3 (48.7–54.3)	0.01
Role physical, median (IQR)	54.9 (47.8–54.9)	47.8 (43.6–54.9)	< 0.001
Bodily pain, median (IQR)	59.1 (51.3–59.1)	59.1 (51.3–59.1)	0.74
General health, median (IQR)	52.7 (52.7–60.0)	52.7 (43.0–52.7)	0.02
Vitality, median (IQR)	55.5 (47.4–55.5)	47.4 (47.4–55.5)	< 0.001
Social functioning, median (IQR)	55.2 (47.3–55.2)	47.3 (41.4–55.2)	< 0.001
Role emotional, median (IQR)	49.0 (49.0–55.2)	43.7 (43.7–49.0)	< 0.001
Mental health, median (IQR)	51.2 (45.1–56.7)	45.1 (38.3–45.1)	< 0.001
PCS, median (IQR)	53.8 (51.0–55.5)	53.6 (50.5–56.6)	0.98
MCS, median (IQR)	50.1 (46.2–54.5)	43.4 (37.6–47.6)	< 0.001

*IQR* interquartile range, *ISI* Insomnia severity index, *MCS* Mental component summary, *PCS* Physical component summary, *PSQI* Pittsburgh sleep quality index, *TST* total sleep time

**Fig. 1:**
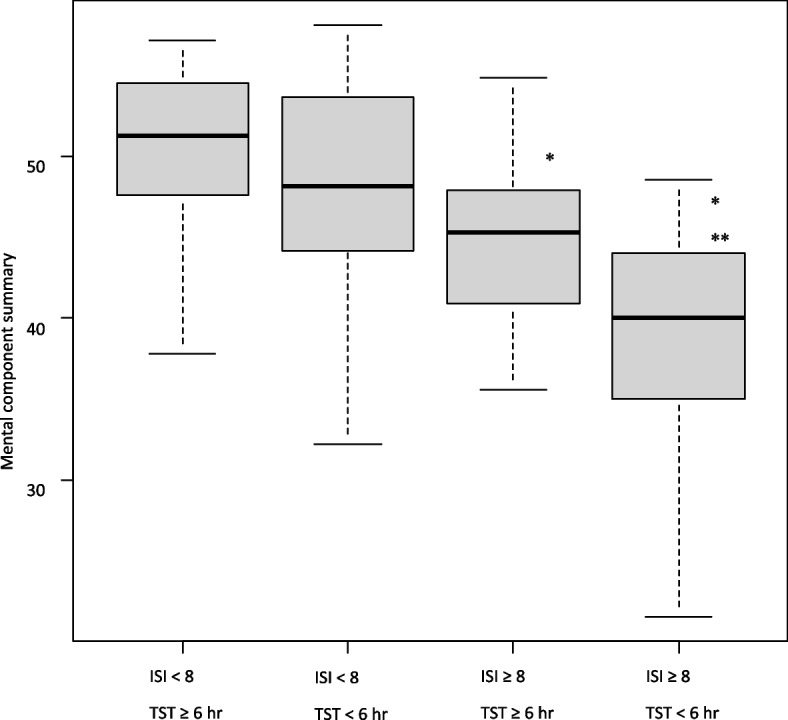
Mental component summary stratified into 4 groups by insomnia severity and total sleep time. * *vs*. ISI < 8 & TST ≥ 6 h group, *P* < 0.001. ** *vs*. ISI < 8 & TST < 6 h group, *P* < 0.001. ISI: Insomnia severity index, TST: total sleep time

As sleep problems were found to affect the decline in HRQOL, especially MCS, the median MCS was used to divide the subjects into two groups (MCS < 48 and MCS ≥ 48), and factors that affected MCS decline were examined. When adjusted by sex, year level, smoking habits, and habitual drinking, ISI ≥ 8 and TST < 6 h on weekdays were significant related factors (Table 4). Subsequently, the participants were divided into four groups using ISI and TST (ISI < 8 & TST ≥ 6 h, ISI < 8 & TST < 6 h, ISI ≥ 8 & TST ≥ 6 h, and ISI ≥ 8 & TST < 6 h). When compared to ISI < 8 and TST ≥ 6 h, MCS was significantly lower if ISI was ≥ 8 and even lower when TST was < 6 h (Fig. 1).

**Table 4:** Logistic regression analysis for poor mental component summary

	Univariable analysis Odds ratio (95% CI)	Adjusted Odds ratio (95%CI)^a^
Weekday total sleep time		
< 6 h	2.62 (1.14–6.04)	3.38 (1.40–8.17)
6–7 h	1	1
≥ 7 h	1.32 (0.57–3.07)	1.62 (0.69–3.82)
Chronotype		
Morning type	1	
Evening type	1.83 (0.92–3.65)	
Insomnia severity index		
< 8	1	1
≥ 8	8.97 (3.64–22.10)	6.51 (2.96–14.30)
Catch-up on sleep		
< 2 h	1	
≥ 2 h	1.00 (0.52–1.92)	

*CI* confidence interval

^a^Adjusted for sex, third or fourth year in university, smoking, and habitual drinking

## Discussion

A survey of third and fourth-year students in nursing colleges revealed that insomnia and short sleep time were associated with mental health decline. The coexistence of insomnia and short sleep caused the most significant decrease in mental health. In general, insomnia patients with short sleep time of < 6 h are the insomnia subtype with the most problems [28]. In this study of young adults as well, the group suspected of this insomnia subtype had low mental health. Hence, it is necessary to pay attention to the complications of mental illness, such as depression, the impact on student life, and the subsequent employment. Improved sleep quality has been shown to lead to better mental health [29], and appropriate intervention is desired.

Most study participants had longer sleep hours on weekends than weekdays, and about half of them slept ≥ 2 h longer on weekends. This catch-up sleep was not associated with a decrease in HRQOL. Catch-up sleep has been reported to be associated with good HRQOL in adults, decreased body mass index, low risk of metabolic syndrome, and low risk of depression [30–33]. Based on the previous reports, the participants were subdivided by catch-up sleep time and examined, but no association with HRQOL including mental health was found. It is possible that insufficient sleep on weekdays was so severe that short catch-up sleep on one or two days off did not have a sufficient effect.

Prolonged sleep time for a longer period of time has been reported to reduce daytime sleepiness, improve mental symptoms, such as depressive symptoms; and improve the athletic performance of college athletes [34–36]. Furthermore, it has been reported that ensuring sufficient sleep time on a regular basis puts an end to the subsequent decline in performance during sleep restriction [37]. As such, even if a person is feeling nervous and is unable to sleep, this banking sleep effect may maintain performance to such a level that attenuates the effects of insomnia, if the person has had enough sleep up until that point in time. In the future, interventions that can extend sleep time are needed.

This study needs to consider the following limitations. First, as parameters like sleep time are evaluated by subjective time, it would be desirable to conduct objective measurement. The aforementioned insomnia subtypes also reveal the problem of short sleep insomnia based on objective sleep time [28, 38–40]. In the future, to evaluate the objective sleep time, it is necessary to examine the presence of arousal reaction and the deep sleep time and ratio together with the electroencephalography. We examined the relationship between HRQOL and various sleep–wake problems in this study, but the relationship with more specific mental and physical problems remains unclear. Furthermore, the relationship between sleep–wake problems and tardiness/absenteeism and grades [14–16] are yet to be clarified. For newly graduated nurses, it is also necessary to consider the relationship between such sleep problems and leave of absence as well as turnover after employment [23, 24].

If insomnia is accompanied by short sleep duration, it is presumed to be a group that requires intervention, which may cause problems not only in schoolwork, but also in subsequent employment. If it is a relatively mild problem, it may be possible to address the issue by student education, including sleep hygiene guidance and self-care advice based on screening results. However, it is assumed that the group with insomnia and short sleep duration will require medical treatment because they are part of a more serious group, which requires consultation recommendations, among other measures. In insomniacs with short sleep duration, the above-mentioned prolongation of sleep duration may cause insomnia symptoms to manifest. They require an approach that includes cognitive-behavioral therapy for insomnia.

Insomnia and short sleep duration were found to be associated with decreased mental health among nursing college students. Additionally, many students tended to lack sleep on weekdays. It is required to comprehensively address various sleep–wake problems identified while in university.

## Data Availability

The datasets used and/or analyzed during the current study are available from the corresponding author on reasonable request.
